# Granulocyte Colony-Stimulating Factor Utilization and Prescribing Patterns in Cancer Patients: A Single Institution Experience of a Saudi Cancer Center

**DOI:** 10.7759/cureus.27017

**Published:** 2022-07-19

**Authors:** Mohammad J Alyamani, Haya AlSalloum, Ghada Elgohary, Khalid Alsaleh, Ahmed Abd El Warith, Nashwa Abd El-Aziz

**Affiliations:** 1 Diabetes and Endocrinology, College of Pharmacy, AlMaarefa University, Riyadh, SAU; 2 Department of Clinical Pharmacy, King Saud University Medical City, Riyadh, SAU; 3 Department of Medical Oncology, King Saud University Medical City, Riyadh, SAU; 4 Department of Internal Medicine and Clinical Hematology, Faculty of Medicine Ain Shams University, Cairo, EGY; 5 Medical Oncology, King Saud University Medical City, Riyadh, SAU; 6 Cancer Insitute, Cairo University, Cairo, EGY; 7 Department of Medical Oncology, King Saud University, Riyadh, SAU; 8 Department of Medical Oncology, South Egypt Cancer Institute - Assiut University, Assiut, EGY

**Keywords:** cancer therapy, csf, g-csf, chemotherapy, febrile neutropenia

## Abstract

Background

Febrile neutropenia (FN), owing to its negative association with immune function and infectious complications, acts as a treatment-limiting factor in myelotoxic cancer chemotherapy. This study aimed to analyze the incidence of FN, utilization of granulocyte colony-stimulating factor (G-CSF) in patients who experienced FN, and its association with age and comorbidities.

Methodology

This retrospective study was conducted in a major tertiary hospital in Riyadh, Kingdom of Saudi Arabia. Inclusion criteria entailed all neutropenic adults aged >18 years with a proven cancer diagnosis, including solid and hematological malignancies. Patients who were treated with chemotherapy and G-CSF were included in the study. Data regarding FN, administration of G-CSF, and patient and physician-related factors were collected.

Results

We collected data on 53 cancer patients with a mean age of 41.9 ± 17.1 years (range = 16-75). FN was present in 16 (30.2%) patients and absent in 37 (69.8%) patients. The mean neutrophil count post-filgrastim did not significantly differ from pre-chemotherapy neutrophil count (Student’s t-test, p = 0.067), while there was a significant difference from post-chemotherapy neutrophil count (Student’s t-test, p = 0.044). In our cohort, 24 (45.3%) patients achieved remission, 12 (22.6%) died, and 17 (32.1%) were not cured. We did not find any significant association between gender, specialty, comorbidities, and age with FN.

Conclusions

G-CSF administration significantly decreases the incidence of FN post-chemotherapy in cancer patients.

## Introduction

With subsequent infectious complications, neutropenia represents one of the common treatment-limiting factors in myelotoxic cancer chemotherapy [1,2]. Febrile neutropenia (FN) is the development of fever, often with other signs of infection, in a patient with neutropenia, which refers to an abnormally low number of neutrophils in the blood [3]. Common chemotherapy regimens lead to FN in 25-40% of treatment-naive patients, resulting in compromised immune function with an increased risk of infections [4]. FN may lead to subsequent dose reduction or delay in chemotherapy, resulting in poor patient outcomes, reduced quality of life, increased length of hospital stay, and higher diagnostic and treatment costs [5-8]. Not only does FN affect individual patient outcomes it also causes a perturbance of resource management according to the pharmacoeconomics principle [9]. Age, comorbidity, and severity of the myelotoxic effect of chemotherapy are the main determinants of the mortality rate associated with FN, ranging from 5-13.7% [10], while the severity of FN depends on the dose intensity of the chemotherapy and the patient’s comorbidities and history of either radiation therapy or cytotoxic treatment.

Colony-stimulating factors (CSFs) such as granulocyte colony-stimulating factor (G-CSF) and granulocyte-macrophage colony-stimulating factor (GM-CSF) act as accelerators of neutrophil production from progenitor cells [11-13]. These adjunctive agents are an integral part of the preventive treatment plans for chemotherapy-induced FN and successfully reduce the severity and duration of FN [14-16]. Filgrastim has been approved by the Food and Drug Administration (FDA) for use in oncology to prevent therapy-induced anemia and neutropenia post-chemotherapy [4]. The American Society of Clinical Oncology (ASCO), in their prevalent guidelines, recommends primary prophylaxis, with CSFs being confined to patients with ≥20% risk of FN [4]. Due to the acceptance of the guidelines profile of filgrastim, 20 million patients have benefited worldwide [17].

Despite the guidelines and recommendations for administering these agents [18-20], the clinical application remains inconsistent [13,21]. Bennett et al. reported on the adherence inconsistency of G-CSF administration, characterized by overutilization in low-risk patients and underutilization in high-risk patients, with 96% of all G-CSF administrations not adhering to the current guidelines [2]. Further studies are warranted to optimally define the patient population that is most likely to benefit from G-CSF therapy [22]. Due to the small sample size with a lack of statistical power, clinical trials have not yet established a significant impact on overall or disease-free survival [11,13]. This study aimed to evaluate the clinical prescribing patterns of G-CSF; identify overuse, underuse, and misuse rates; and identify patient and physician factors associated with such utility.

## Materials and methods

After acquiring appropriate Institutional Review Board approval, a retrospective study was conducted at King Khalid University Hospital’s Oncology Center, Riyadh, Kingdom of Saudi Arabia following the ethical principles based on the Declaration of Helsinki (2013), consistent with the Good Clinical Practice ICH Tripartite guideline [23]. Data were collected for all neutropenic adults aged >18 years with a proven diagnosis of hematological and solid malignancies who were treated with chemotherapy and received G-CSF as primary or secondary prophylaxis. The data collected included risk factors for starting G-CSF as primary or secondary prophylaxis, the timing of G-CSF usage with chemotherapy lagging period, number of doses needed to attain absolute neutrophil count (ANC) levels of >500 cell/µL for three consecutive days, neutrophilic type, and count follow-up, as well as hospitalization and fever duration.

Statistical analysis

Descriptive statistics were computed as baseline means, standard deviations (SDs), minimum and maximum values for continuous variables, and percentages for categorical variables. The chi-square and Fisher exact test were used for comparing FN to gender, specialty, comorbidity, and age. Student’s t-test was used to compare neutrophil count pre-and post-chemotherapy and with post-filgrastim. We used STATA version 13.0 (Stata Corp., College Station, TX, USA) for analyzing data. A statistical significance threshold of p < 0.05 was adopted.

## Results

We found complete data on 53 cancer patients who fulfilled our inclusion criteria with a mean age of 41.9 ± 17.1 years (range = 16-75) who were on myelosuppressive regimens. Treatment objectives were curative in 49 (92.4%) and palliative in four (7.5%) patients. The specialty was hematology in 34 (64.1%) and oncology in 19 (35.8%) patients.

FN was present in 14 (26.4%) and absent in 39 (73.6%) patients. Bacterial culture was positive in nine (16.9%) patients. Antibiotic was received by 20 (35.7%) patients, among which antibiotic was stopped because the culture was negative in six (30%) patients, while the course was completed in 14 (70%) patients. Among our cohort, 24 (45%) patients achieved remission, 12 (23%) died, and 17 (32%) patients were not cured.

Pre-chemotherapy, the mean neutrophil count was 4.8 ± 4.8 (range = 0.1-24.2); post-chemotherapy, the mean neutrophil count was 3.1 ± 3.2 (range = 0-18.3); and post-filgrastim, the neutrophil count was 9.4 ± 6.8 (range = 0.7-33). The mean neutrophil count post-filgrastim did not significantly differ from pre-chemotherapy count (Student’s t-test, p = 0.067), while it significantly differed from post-chemotherapy neutrophil count (Student’s t-test, p = 0.0247).

Table 1 summarizes the correlation between FN and gender, specialty, comorbidities, and age; however, none of the variables were significantly correlated with FN. Table 2 shows the frequency of cancer subtypes.

**Table 1:** Correlation between febrile neutropenia with demographics, comorbidities, and other factors with Fisher exact and chi-square p-values. SD: standard deviation; CI: confidence interval

Variables	Febrile neutropenia		Total	P-value
	Yes	No		
Gender				
Female	4 (28.6)	18 (46.2)	22 (41.5)	0.25
Male	10 (71.4)	21 (53.8)	31 (58.5)	
Specialty				
Hematology	10 (76.9)	23 (59.0)	34 (64.15)	0.24
Oncology	3 (23.1)	16 (41.0)	19 (35.84)	
Comorbidities				
None	8 (57.1)	27 (69.2)	35 (66.0)	0.44
Hypertension and diabetes	0 (0)	1 (2.6)	1 (1.9)	
Diabetes	2 (14.3)	7 (17.9)	9 (17.0)	
Others	4 (28.6)	4 (10.3)	8 (15.1)	
Age				
	40.7 ± 16.8 (CI: 31-50)	42.8 ± 17.4 (CI: 37-48)		0.69
Neutrophil count	Pre-chemotherapy	Post-chemotherapy		
Mean (SD)	7.4 (7.5)	4.69 (3.9)	34	0.0247

**Table 2:** Frequency of cancer subtypes. ALL: acute lymphoblastic leukemia; AML: acute myeloid leukemia; CLL: chronic lymphocytic leukemia; DLBCL: diffuse large B-cell lymphoma; FL: follicular lymphoma; MCL: mantle cell lymphoma; MM: multiple myeloma; NHL: non-Hodgkin’s lymphomas; RCC: renal cell carcinoma

Cancer diagnosis	Frequency	Percentage	Cumulative
Urothelial cancer	1	1.85	1.85
ALL	4	7.54	11.11
AML	3	5.56	16.67
Breast cancer	8	14.81	31.48
CLL	1	1.85	33.33
DLBCL	6	11.11	44.44
Ewing sarcoma	2	3.7	48.15
Hodgkin’s	8	14.81	62.96
MCL	1	1.85	64.81
MM	1	1.85	66.67
NHL	4	7.41	74.07
RCC	1	1.85	75.93
Relapsed FL	1	1.85	77.78
Skin cancer	1	1.85	79.63
Intestinal	4	7.41	87.04
Lymphoma	5	9.26	96.3
Met *seminoma*	1	1.85	98.15
Uterine sarcoma	1	1.85	100

## Discussion

Cytotoxic chemotherapy-induced FN contributes to a significant increase in morbidity and mortality rates due to compromised immune function and subsequent complications such as fungal infections, Gram-negative sepsis, pneumonia, other lung diseases, cerebrovascular disease, and disorders of the liver and kidney [1,19,24]. G-CSF is approved by the FDA for instigating the production of neutrophils and has been found beneficial in reducing mortality and morbidity [14,20]. However, the data regarding the utility of G-CFS is scarce, and there is a need to elaborate on its therapeutic effect, as well as the effect of patient and physician factors on G-CSF utility. This study aimed to explore the impact of associated physician and patient factors such as physicians’ specialty, patients’ age, gender, and comorbidities with FN, along with the effect of G-CSF on pre- and post-chemotherapy on FN. The adherence to the guidelines has been assessed.

This study showed that physicians’ specialty and patients’ age, gender, and comorbidities had no significant correlation with FN. Contrary to our results, Aapro et al. showed that age is a predisposing factor for FN [18]. Whereas Shaqul et al. did not find any association between age with FN, which is in line with our findings [25]. These disparities highlight the need for further studies. In our cohort of 54 cancer patients, the mean neutrophil count post-filgrastim did not differ significantly from pre-chemotherapy neutrophil count (Student’s t-test, p = 0.067), while there was a significant difference from post-chemotherapy neutrophil count (Student’s t-test, p = 0.044).

Similarly, Shauq et al. recommended using G-CSF and found that it decreases the incidence and severity of neutropenia in breast cancer patients [25]. G-CSF administration significantly reduces the incidence of FN post-chemotherapy in cancer patients. A meta-analysis of 148 studies found a significant reduction in infections with G-CSF used as primary prophylaxis but did not significantly decrease infection-related mortality [16]. In secondary prophylaxis, where it is given prophylactically due to previous FN, G-CSF reduced the time for neutrophil recovery, the incidence of FN, hospitalization, and the administration of antibiotics [9]. While secondary prophylaxis is beneficial, studies comparing primary to secondary prophylaxis tend to support using the former over the latter [12]. However, the cost associated with G-CSFs is another limiting factor that contributes significantly to the overall cost of cancer-related healthcare [9,26,27]. The cost-effectiveness of G-CSF prophylaxis relies on individuals’ risk of developing FN in cancer patients [1,28-30]. Recent economic analyses have indicated that if FN risk is 17-20%, G-CSFs can be a cost-efficient therapy.

The small number of cases in our cohort limited a more detailed statistical analysis or generalization of the findings. However, it identified the use of G-CSF in our institution from where the cases were extracted.

## Conclusions

G-CSF administration is beneficial in reducing morbidity and mortality rates associated with FN. We did not find any association between patients’ associated factors such as age, gender, comorbidities, and incidence of FN. Results of the current study confirmed earlier findings on the therapeutic efficiency of G-CSF in the reduction of FN post-chemotherapy, whereas no significant effect was observed on the incidence of pre-chemotherapy FN.
